# A functional connectome phenotyping dataset including cognitive state and personality measures

**DOI:** 10.1038/sdata.2018.307

**Published:** 2019-02-12

**Authors:** Natacha Mendes, Sabine Oligschläger, Mark E. Lauckner, Johannes Golchert, Julia M. Huntenburg, Marcel Falkiewicz, Melissa Ellamil, Sarah Krause, Blazej M. Baczkowski, Roberto Cozatl, Anastasia Osoianu, Deniz Kumral, Jared Pool, Laura Golz, Maria Dreyer, Philipp Haueis, Rebecca Jost, Yelyzaveta Kramarenko, Haakon Engen, Katharina Ohrnberger, Krzysztof J. Gorgolewski, Nicolas Farrugia, Anahit Babayan, Andrea Reiter, H. Lina Schaare, Janis Reinelt, Josefin Röbbig, Marie Uhlig, Miray Erbey, Michael Gaebler, Jonathan Smallwood, Arno Villringer, Daniel S. Margulies

**Affiliations:** 1 Max Planck Research Group for Neuroanatomy & Connectivity, Max Planck Institute for Human Cognitive and Brain Sciences, Leipzig, Germany; 2 International Max Planck Research School NeuroCom, Leipzig, Germany; 3 Faculty of Biosciences, Pharmacy and Psychology, University Leipzig, Leipzig, Germany; 4 Neurocomputation and Neuroimaging Unit, Department of Education and Psychology, Freie Universität Berlin, Berlin, Germany; 5 Database management, Max Planck Institute for Human Cognitive and Brain Sciences, Leipzig, Germany; 6 Department of Psychology, Technische Universität Dresden, Dresden, Germany; 7 Department of Neurology, Max Planck Institute for Human Cognitive and Brain Sciences, Leipzig, Germany; 8 MindBrainBody Institute, Berlin School of Mind and Brain, Humboldt-Universität zu Berlin, Berlin, Germany; 9 Max Planck Research Group Cognitive and Affective Control of Behavioural Adaptation, Max Planck Institute for Human Cognitive and Brain Sciences, Leipzig, Germany; 10 Universitäre Psychiatrische Kliniken Basel, Basel, Switzerland; 11 Department of Social Neuroscience, Max Planck Institute for Human Cognitive and Brain Sciences, Leipzig, Germany; 12 MRC Cognition and Brain Sciences Unit, Cambridge University, Cambridge, UK; 13 Department of Psychology, Stanford University, Stanford, California 94305, USA; 14 LabSTICC-IMT Atlantique-campus de Brest, Brest, France; 15 Lifespan Developmental Neuroscience, Department of Psychology, Technische Universität Dresden, Dresden, Germany; 16 Department of Psychology, University of York, York, UK

**Keywords:** Functional magnetic resonance imaging, Brain imaging, Human behaviour, Personality

## Abstract

The dataset enables exploration of higher-order cognitive faculties, self-generated mental experience, and personality features in relation to the intrinsic functional architecture of the brain. We provide multimodal magnetic resonance imaging (MRI) data and a broad set of state and trait phenotypic assessments: mind-wandering, personality traits, and cognitive abilities. Specifically, 194 healthy participants (between 20 and 75 years of age) filled out 31 questionnaires, performed 7 tasks, and reported 4 probes of in-scanner mind-wandering. The scanning session included four 15.5-min resting-state functional MRI runs using a multiband EPI sequence and a hig h-resolution structural scan using a 3D MP2RAGE sequence. This dataset constitutes one part of the MPI-Leipzig Mind-Brain-Body database.

## Background & Summary

Understanding the unique features of brain organization giving rise to distinct patterns of behavior, cognition, and mental experience remains one of the key research questions in the emerging field of human functional connectomics^1^. Functional connectivity has become a prominent method for investigating phenotypic differences across individuals^2,3^. However, there is ever greater need for validation of findings across independent datasets. The dataset presented here joins several others in contributing to this research agenda^4–6^ (Data Citation 1) and provides an additional resource for cross-site validation studies.

We acquired a wide range of self-reported personality measures as well as features of self-generated mental experience. In addition, a core magnetic resonance imaging (MRI) dataset—including one-hour of resting-state functional MRI (rs-fMRI) data—was acquired on 194 healthy participants. Questionnaires and behavioral measures were acquired over several follow-up sessions.

This dataset constitutes one part of the MPI-Leipzig Mind-Brain-Body (MPILMBB) database, which consists of data from a partially overlapping cohort of participants^7^. The contribution described here enables exploration of individual variance across cognitive and emotional phenotypes in relation to the brain, which is complemented by data regarding physiology, clinical assessment, and anthropometric measures described in our related publication^7^. All MRI data across the MPILMBB were acquired on the same Siemens Verio 3 Tesla MRI scanner.

## Methods

### Participants

**Figure 1:**
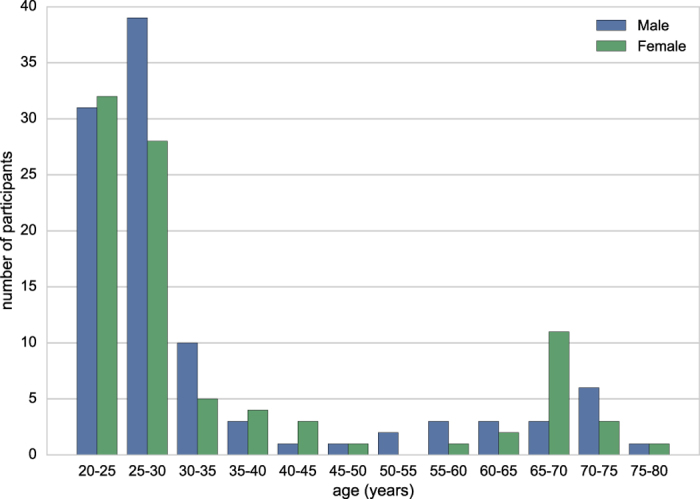
Age distribution. Age distribution (5-year bins) of the participants split by gender.

In total, datasets from 194 native German-speaking participants are included (94 female, mean age=34 years, median age=27, SD=16 years; Fig. 1; see Supplementary Table 1 and Supplementary File 1). All participants were scanned on a 3 Tesla magnetic resonance imaging (MRI) scanner (Siemens Magnetom Verio) for the acquisition of one structural and four rs-fMRI scans. In addition, extensive questionnaire and task performance data were acquired from each participant. A subset of participants (N=109) were also included in a complementary data acquisition.

#### Recruitment and inclusion criteria

Prospective participants were initially recruited by the Leipzig Study for Mind-Body-Emotion Interactions project. Additional participants were recruited through online and poster advertisements. All participants were prescreened via telephone to determine their eligibility for the current study (Box 1). Participants fulfilling the eligibility criteria (including medical screening for MRI-scanning and neurological history) were invited to Max Planck Institute for Human Cognitive and Brain Sciences (MPI-CBS) where they were screened for past and present psychiatric disorders using the Structured Clinical Interview for DSM-IV (SCID-I^8^). After meeting eligibility criteria, participants received detailed information regarding the study.

All participants fulfilled the MRI safety requirements of the MPI-CBS (Supplementary Table 2), provided written informed consent (including agreement to their data being shared anonymously) prior to their participation in the study. Participants received monetary compensation for their participation. The study protocol was approved by the ethics committee at the medical faculty of the University of Leipzig (097/15-ff).

### Data acquisition and protocol overview

Participants were required to complete: 1) four functional MRI scans within one scanning session and, if not previously acquired, one structural scan; 2) a battery of personality and mind-wandering questionnaires spread over five appointments, and 3) a set of cognitive control and sustained attention, synesthesia, and creativity tasks spread over two appointments.

The data acquisition took place over five appointments over a two-year period (see Table 1):

- Day 1: We acquired data on a set of questionnaires that were completed at MPI-CBS (Table 1).
- Day 2: We sent personalized links to participants, who could complete the set of online questionnaires at their convenience (Table 1).
- Day 3: Participants were scanned at the Day Clinic for Cognitive Neurology, University of Leipzig. Before entering the scanner, participants completed a pen-and-paper practice trial of the short version of the New York Cognition Questionnaire^9^. While in the scanner, and immediately after each of the four resting state runs, participants received the computerized version of the same questionnaire. Immediately after the scanning session participants received additional questionnaires and a set of tasks (Table 1).
- Day 4: The Abbreviated Math Anxiety Scale^10^ and the NEO Personality Inventory-Revised^11–13^ were completed online at the participant’s convenience (Table 1).
- Day 5: We acquired data on a set of questionnaires and tasks that were administered at MPI-CBS. Tasks were conducted using pen-and-paper, computer-administered, as well as Limesurvey (http://www.limesurvey.org; version 2.00+) interfaces (Table 1).

Within each set of questionnaires and tasks, the order of presentation of questionnaires and tasks was randomized across participants. If participants failed to complete a given questionnaire it was excluded from data analysis. Due to dropout, not all participants completed the full set of questionnaires and tasks (Table 2).

### Behavioral measures

Below we provide a short description of the acquired behavioral measures assessing: Personality and habitual behaviors, mind-wandering and mindfulness, synesthesia, cognitive control and sustained attention, and creativity.

### Personality and Habitual Behaviors

#### Abbreviated Math Anxiety Scale (AMAS)

The AMAS is a self-report inventory measuring the subjectively experienced level of anxiety in mathematical contexts^10^. It consists of nine items, related to the question “How anxious do you feel when …”, that can be scored on a five-point Likert scale (1=“not at all” to 5=“a lot”). We used a German translated version of the original English questionnaire.

#### Adult Self Report (ASR)

The ASR assesses mental problems in adults between 18 and 59 years-old^14^. It has four major scales related to the following topics: adaptive functioning, psychological syndromes, DSM-oriented problems, and substance use. Adaptive functioning comprises 36 items in the form of either a three or four-point Likert scale describing the quantity and quality of relationships, education level, and job satisfaction. Comments to open questions are not made openly available. Scales of psychological syndromes, DSM-oriented problems, and substance use comprise 126 items that can be scored on a three-point Likert scale (0=“does not apply” to 2=“exactly or does happen often”). Two items were erroneously excluded (i.e., item 56.h “Heart pounding or racing”; item 56.i “Numbness or tingling in body parts”). These affect somatic complaints and internalizing subscales of the psychological syndromes scale. We used the German ASR version^14^.

#### Beck Depression Inventar-II (BDI)

The BDI-II measures the severity of various depressive symptoms in adolescents and adults over the two weeks prior to completion of the inventar^15,16^. It consists of 21 items that require multiple-choice answers that best describe statements about subjectively experienced states. The items can be scored on a four-point Likert scale (e.g., 0=“I do not feel sad.” to 3=“I am so sad or unhappy that I can't stand it”). We used the German BDI version^17^.

#### Behavioral Inhibition and Approach System (BIS/BAS)

The BIS/BAS^18^ measures individual differences in response to two motivational systems: behavioral inhibition and behavioral approach (systems postulated by Gray^19,20^). It comprises a total of 24 items that can be scored using a four-point Likert-type scale (1=“not true for me at all” to 4=“very true for me”). We used the German version of the questionnaire^21^.

#### Body Consciousness Questionnaire (BCQ)

The BCQ assesses three components of body consciousness: private body (e.g., heartbeat perception), public body (perception of outward appearance), and body competence (aspects of the body, e.g., strength)^22^. The questionnaire consists of 15 items that can be scored on a five-point Likert scale (0=“extremely uncharacteristic” to 4=“extremely characteristic”). We used a German translated version of the original English questionnaire.

#### Boredom Proneness Scale (BP)

The BP measures the tendency to experience boredom, in particular the self-reported lack of internal and external stimulation^23^. It consists of 28 items that can be scored on a seven-point Likert scale (1=“total disagreement” to 7=“total agreement”). We used a German translated version of the original English scale.

#### Brief Self-Control Scale (SCS)

The SCS is a self-report measurement assessing the capacity for self-control^24^. Self-control was operationalized as the capability to modify or override one's own response tendencies^24^. We used the German adaption of the brief SCS^25^. It consists of 13 items that can be scored on a five-point Likert scale (1=“do not agree at all” to 5=“completely agree”).

#### Epworth Sleepiness Scale (ESS)

The ESS measures tendencies of sleepiness in everyday life^26^. The scale consists of eight items addressing the subjective propensity to fall asleep in different situations. The items can be scored on a four-point Likert scale (0=“would never doze” to 3=“high chance of dozing”). We used the German ESS version^27^.

#### Facebook Intensity Scale (FBI)

The FBI measures the intensity of Facebook usage that incorporates emotional connectedness to the site, its integration into daily activities, membership duration, and the number of friends^28^. It consists of eight items that can be scored on a five-point Likert scale (1=“strongly disagree” to 5=”strongly agree”). Small alterations in the formulation and in the order of presentation of the items were applied—see the ^∗^.txt file of this questionnaire. We used a German translated and adapted version of the original English scale.

#### Goldsmiths Musical Sophistication Index (Gold-MSI)

The Gold-MSI measures the level of experience with and understanding of music in community samples^29^. A subset of 16 items was measured, including the active engagement subscale and the musical training subscales (the item order is explained in the ^∗^.txt file of this index). The subscales perceptual abilities, singing abilities, and emotions were not included in the measurement. The items can be scored on a seven-point Likert scale (1=“completely disagree” to 7=“completely agree”). We used the German version of the index^30^.

#### Hospital Anxiety and Depression Scale (HADS)

The HADS measures the severity of depression- and anxiety-related symptoms^31^ for the week prior to completion and can be used to assess subclinical tendencies of depression and anxiety. It consists of 14 items in total that can be scored on a four-point Likert scale (e.g., 1=“most of the time” to 4=“never”). We used the German HADS version^32^.

#### Internet Addiction Test (IAT)

The IAT assesses self-reported excessive use of the Internet^33^. The test is comprised of 20 items that can be scored on a six-point Likert scale (0=“does not apply” to 5=“always”). We used item three (i.e., “how often do you prefer the excitement of the Internet to intimacy with your partner?”) with a different scale compared to the original one. Therefore, this item was not included in the scoring of the scale. We used a German translated and adapted version of the original English test.

#### Involuntary Musical Imagery Scale (IMIS)

IMIS is a self-report inventory measuring phenomenological properties of the experiential tendency of having involuntary musical imagery, also known as “earworms”^34^. It measures four facets of involuntary musical imagery: the subjective evaluation of this phenomenon (negative valence), the embodied responses (movement), the personal contemplations (personal reflections), and the constructive properties (help). It consists of 18 items that can be scored on different scales: 14 items can be scored on a five-point Likert scale (1=“never” to 5=“always”); two items with different five-point Likert scales (e.g., 1=“less than 5 seconds” to 5=“more than a minute”); one item with a six-point Likert scale (1=“never” to 6=“almost continuously”). The English questionnaire consists of two parts (A and B) which were combined in the German version (see the respective ^∗^.txt file for more details). We used a German translated and adapted version of the original English scale.

#### Mobile Phone Usage (MPU)

This in-house developed collection of items measures various patterns of mobile phone usage, such as e-mail usage as well as the use of social network sites via smartphone. It consists of 19 items with various answer formats. A translated version of the original English questionnaire (see below) was used.

1. Do you own a mobile phone? (yes, no)
2. How often do you have your mobile phone on you?
3. all the time
4. most of the day
5. a few hours a day
6. How many text messages do you send a week (on average)?
7. How many phone calls (using your mobile phone) do you make/take per week (on average)?
8. Do you own a smartphone (iPhone, Android etc. - a multipurpose phone with Internet connection)? (yes, no)
9. Do you have a flat Internet rate? (yes, no)
10. How long have you been using a smartphone [months, years…]?
11. Do you use your smartphone to browse the web? (yes, no)
12. How often do you look up facts on your phone in a social situation?
13. never
14. sometimes
15. often
16. Do you use your smartphone to check your email? (yes, no)
17. Do you get a notification each time you get a new email or do you have to check it manually?
18. If manually, how often?
19. never
20. sometimes
21. often
22. Do you use your phone for social networking (Facebook, Twitter, Google+ etc.)?
23. never
24. sometimes
25. often
26. Do you post messages (including tweets) to social networks using your phone (as opposed to just reading)?
27. Do you use your phone for instant messaging (Viber, WhatsApp, Google chat, Facebook Messaging etc.)?
28. never
29. sometimes
30. often
31. Do you use your phone to check the news?
32. never
33. sometimes
34. often
35. Do you use your phone to take and share pictures?
36. never
37. sometimes
38. often
39. In a social situation such as a dinner with friends how often do you have an urge to check your phone (to check the news, Facebook etc.)?
40. never
41. sometimes
42. often
43. How often do you resist this urge due to social pressure?
44. never
45. sometimes
46. often
47. Do you read printed newspapers/magazines?
48. never
49. sometimes
50. often
51. How many books do you read a year?

#### Multi-Gender Identity Questionnaire (MGIQ)

The MGIQ used in this study was adapted from the Multi-Gender Identity Questionnaire developed by Joel and colleagues^35^. The questionnaire assesses: gender identity, sexual orientation, gender of the preferred sexual partner, preferred form of relationship, and attitudes towards the social construction of gender. The original questionnaire consists of 38 items that can be scored on either a five- or a six-point Likert scale (1=“always” to 5=“never”; 1=“always” to 6=“does not apply”). Two additional sections—relevance of the MGIQ questions and demographic details—were added to the questionnaire. Please see the respective ^∗^.txt file for details on the modifications and additional sections. We used a German translated and adapted version of the original English questionnaire.

#### Multimedia Multitasking Index (MMI)

The MMI measures the extent of simultaneous use of 12 different media types^36^: computer-based streaming (video, music), non-music audio, computer games, voice calls, instant messaging, text messaging, email, web surfing, and other applications such as Word processing. It consists of a total of 219 items, across the 12 media types, that can be scored on different Likert scales (e.g., 1=“never” to 4=“most of the time”; 1=“more time” to 3=“same amount of time”). We used a translated version of the original English index.

#### NEO Personality Inventory-Revised (NEO PI-R)

The NEO PI-R assesses the five personality traits: extraversion, agreeableness, conscientiousness, neuroticism, and openness to experience^11,12^. Moreover, the questionnaire also assesses six underlying facets for each of the five main factors. It consists of 241 items that can be scored on a five-point Likert scale. We used the German version of the inventory^12^. Due to a technical error, item 71 (i.e., “I am seldom sad or depressed”) was measured twice; one time instead of item 46 (i.e., “I seldom feel self-conscious when I'm around people”). Thus, item 46 was not taken into account for the summary score of subscale N3. Additionally, item 83 was missing and was therefore not taken into account for creating subscale O5.

#### Personality Style and Disorder Inventory (PSSI)

The PSSI is a self-report measurement assessing 14 personality styles^37^. These personality styles are conceptualized as non-pathologic, sub-clinical equivalents of personality disorders as described in diagnostic manuals such as the Diagnostic and Statistical Manual of Mental Disorders^38^. The inventory consists of 140 items that can be scored on a four-point Likert scale (1=“do not agree” to 4=“highly agree”).

#### Self-Esteem Scale (SE)

The SE is a self-report scale measuring global self-worth by assessing positive and negative feelings about the self^39^. It comprises eight items that can be scored on a six-point Likert scale (0=“does not apply” to 5=“applies to me”). We used a German translated version of the original English scale.

#### Short Dark Triad (SD3)

The SD3 assesses the following personality traits: machiavellianism, narcissism, and psychopathy in their subclinical manifestations^40^. It consists of 27 items that can be scored on a five-point Likert scale (1=“strongly disagree” to 5=“strongly agree”). The questionnaire was retrieved from an online platform (http://www.midss.org/sites/default/files/d3.pdf) previous to its publication^40^. Thus, item two of the used questionnaire (i.e., “Generally speaking, people won't work hard unless they have to”) is different from the published version (i.e., “I like to use clever manipulation to get my way”). We used a German translated version of the original English questionnaire.

#### Social Desirability Scale-17 (SDS)

The SDS is a self-report questionnaire that assesses one's tendency to seek social approval^41^, and it can be used to control for biased answer’s tendencies due to social desirability. We used a German version of the scale^41^ consisting of 17 items that can be scored on a five-point Likert scale (1=“do not agree at all” to 5=“completely agree”).

#### Tuckman Procrastination Scale (TPS)

The TPS assesses self-reports of procrastination in everyday life, which are related to the tendency to inappropriately delay pending tasks^42^. It consists of 16 items that can be scored on a five-point Likert scale (1=“does not apply to me at all” to 5=“applies to me to a great extent”). We used the German version of the scale (TPS-D; https://www.dgps.de/fachgruppen/diff_psy/pdf/instrumente/Prokrastination.pdf).

#### UPPS-P Impulsive Behavior Scale (UPPS-P)

The UPPS-P is a self-report measure of different trait aspects of impulsive behavior^43,44^. This revised scale^44^ quantifies five distinguishable facets of impulsivity: positive urgency, negative urgency, lack of premeditation, lack of perseverance, and sensation seeking. It consists of 59 items that can be scored on a four-point Likert scale (1=“strongly agree” to 4=“strongly disagree”). We used a German translated version of the original English questionnaire cf.^45^.

### Mind-Wandering and Mindfulness

#### Five Facets of Mindfulness Questionnaire (FFMQ)

The FFMQ assesses five aspects of mindfulness^46^: observation of internal and external processes, description of internal processes, conscious actions, non-judgement about mental processes, and non-reaction to mental processes. It consists of 39 items that can be scored on a five-point Likert scale (1=“never or very rarely true” to 5=“very often or always true”). We used a German translated version of the original English questionnaire.

#### Metacognition Questionnaire (MCQ-30)

The MCQ-30 assesses self-reported attitudes and abilities in relation to: worrying, trust in cognitive abilities, control over thoughts, and cognitive self-consciousness^47^. It consists of 30 items that can be scored on a four-point Likert scale (1=“do not agree” to 4=“agree very much”). The order of presentation of the items was done according to Sadeghi and colleagues^48^. We used a German translated version of the original English questionnaire.

#### New York Cognition Questionnaire (NYC-Q)

The NYC-Q is a self-report questionnaire that retrospectively measures thoughts and feelings experienced by a person while doing a specific task or activity just prior to completion^49^. The NYC-Q consists of two parts. The first part measures the content of thoughts (e.g. past related) and feelings with 23 items. The second part measures the form of thoughts (e.g. in the form of images) with 8 items. The items in both parts of the questionnaire can be scored on a nine-point Likert scale (First part: 1=“completely did not describe my thoughts” to 9=“completely did describe my thoughts”; Second part: 1=“completely does not characterize my experience” to 9=“completely characterize my experience”). We assessed the NYC-Q at two time points: 1) immediately after the scanning session and 2) after both the emotional task switching (ETS)^50^ and conjunctive continuous performance task (CCPT)^51^ were completed. For the NYC-Q presented after both ETS and CCPT, the first part of the questionnaire was consistently assessed; while the second part is only available for a subset of participants.

#### Short Version of the New York Cognition Questionnaire (Short-NYC-Q)

The short-NYC-Q^9^ is similar to the NYC-Q^50^, but it only uses 12 items to measure form and content of mind-wandering. The questions can be rated using a digital format of a scale bar, with an answer resolution of 5% increments (0%=“describes my thoughts not at all”- 100%=“describes my thoughts completely).

#### Spontaneous and Deliberate Mind-Wandering (S-D-MW)

Both spontaneous mind-wandering (S-MW) and deliberate mind-wandering (D-MW) quantify trait-level tendencies to experience spontaneous and deliberate forms of mind-wandering^52^. Each of the scales comprises four items. The D-MW scale captures experiences of intentional mind-wandering, whereas the S-MW scale assesses unintentional occasions of mind-wandering. Although in the original questionnaire the items can be scored on a seven-point Likert scale, we have adopted a five-point Likert scale instead (1=“almost never” to 5=“very often”). We used a German translated version of the original English questionnaire cf.^45^.

#### Varieties of Inner Speech Questionnaire (VISQ)

The VISQ measures phenomenological properties of inner speech^53^. The questionnaire includes 18 items assessing four dimensions of inner speech: dialogic inner speech, the extent to which inner speech involves the voice of others, condensed/expanded inner speech, and evaluative/motivational inner speech^53^. The items can be scored using a six-point Likert scale (1=‘‘Certainly does not apply to me’’ to 6=“Certainly applies to me”). We used a German translated version of the original English questionnaire.

### Synesthesia

#### Synesthesia Color Picker Test (SYN)

The SYN measures the consistency of synesthetic color experiences in response to graphemes (letters and numbers)^54^. Participants assign colors to repeatedly presented graphemes. Digits 0–9 and all letters of the alphabet were randomly repeated three times. Perfect consistency would be reflected in a score of 0. A consistency score of 1 or less indicates the presence of grapheme-color synesthesia.

### Cognitive Control and Sustained Attention

#### Adaptive Visual and Auditory Oddball Target Detection Task (Oddball)

This task was designed to estimate the modality specific (visual/auditory) perceptual threshold in relation to content and form of ongoing thoughts that were experienced during the task. Based on a common “oddball” paradigm e.g.,^55^, participants had to respond via button press to target stimuli—amplitude modulated gabor patches [visual condition], and sinus tone waves [auditory condition]—that occur infrequently and irregularly within a series of standard stimuli. The task was designed to adapt to the level of the participant’s performance, that is, the better the performance, the lower the deviation between the infrequent and standard stimuli (1-up 2-down staircase procedure). From time to time, participants were interrupted and asked to rate what they had thought about prior to the interruption cf.^56^ by using a visual analogue scale. Visual and auditory conditions appeared to be in two alternating blocks, with 30 deviants per block, 3—7 standard stimuli before each deviant, and 5 thought probes per block. The task had a duration of 60 min.

#### Attention Control Scale (ACS)

The ACS is a self-report inventory constructed to assess individual differences in attentional control^57^. It consists of 20 items that can be scored using a four-point Likert scale (1=“almost never” to 4=“always”). We used a German translated version of the original English scale.

#### Conjunctive Continuous Performance Task (CCPT)

The visual variant of the CCPT^51^ was used to assess the sustained and selective attention of participants. Participants were instructed to accurately and quickly respond to a target stimuli (red square) that infrequently appeared within a series of other geometrically shaped and colored stimuli (e.g., yellow triangle, blue square, etc.). A 4×4×4 design with four geometrical forms, four colors, and four interstimulus intervals was used. Each combination was presented five times, resulting in a total of 320 trials. For demonstration purposes, participants fulfilled a practice round consisting of 15 trials. Immediately after finishing the task, participants completed The New York Cognition Questionnaire (NYC-Q)^49^ to assess several dimensions of thoughts and feelings experienced during the task (see above). The entire procedure lasted 15 min.

#### Emotional Task Switching Task (ETS)

The ETS measures cognitive control, more specifically task switching ability and cognitive inhibition^50,58^. Participants were presented with a series of words and were asked to judge their emotional valence (positive/negative), color (blue/green), or word class (adjective/noun). Participants indicated their response by pressing a button on the left or right side of a word, which corresponded to a congruent forced-choice. There were two blocks with a short pause in between. In total there were 300 trials across the three conditions (i.e., 100 words per category). The order of presentation of the conditions was randomized. The experiment has both N-1 and N-2 trial effects, stemming from either simple task switching (N-1) or task-set inhibition (N-2). The task had a duration of 25 min.

### Creativity

#### Alternative Uses Task (AUT)

The AUT is a measure of divergent thinking^59^. Participants were asked to generate novel and creative uses for three items: an umbrella, a car tire, and a water hose. For each of these items, two minutes were given to generate and write down the ideas. Afterwards, participants had to select and mark their top two answers^60^. Three trained judges rated the answers with respect to (i) creative quality and (ii) amount of detail given (elaboration). The interrater reliability was moderate to high (intra-class correlation of 0.74–0.82) for the rated scores. Further, fluency was assessed, which refers to the total number of given answers per subject. Additionally, the statistical rareness of the answers (originality) was calculated by assessing the relative frequency of each answer. To achieve this, semantically similar answers (e.g. “flower pot” and “plant pot” a use for the car tire) were counted as the same answer.

#### Creative Achievement Questionnaire (CAQ)

The CAQ assesses the amount of creative achievements with 96 items in ten different domains^61^: visual arts, dance, music, drama, culinary arts, architecture, creative writing, humor, science, and invention. Each domain consists of eight ranked questions (e.g., 0=“I do not have training or recognized talent in this area” to 7=“My work has been reviewed in national publications”). We used a German translated version of the original English questionnaire.

#### Remote Associates Test (RAT)

The RAT has mostly been used to operationalize concepts such as creativity or problem solving cf.^62^. The German version of the test consists of 20 word puzzles^63^, presented in counterbalanced sets of ten. Each word puzzle comprises three stimulus words, which seem to be unrelated. Participants are instructed to find out a unifying fourth word that relates to each of the three words. (e.g., work, alarm, ladder - fire). A total of 40 seconds was given for each puzzle (30 seconds thinking time and ten seconds answering time).

#### Test of Creative Imagery Abilities (TCIA)

The TCIA measures creative imagery abilities with the help of a drawing task^64^. Participants are instructed to complete seven ambiguous figures in a creative way. First, participants are asked to generate and write down ideas for completion of the figures. Second, participants have to select one of their ideas and try to illustrate the figure in a way that represents the chosen idea. Finally, a title for the figure needs to be generated. No time limit is given for completion of the task. The drawings were rated by five trained judges in three different categories: (i) vividness, which describes the level of detail and abstraction of the drawing; (ii) originality, which refers to the creative quality in terms of novel and surprising drawings, and (iii) transformativeness, the level of modification and improvement of the initially generated idea. Interrater reliability for those scores was between acceptable and good (intra-class correlation 0.73–0.76).

### Drug screening prior to MRI data acquisition

Each of the participants was instructed not to use illicit drugs within two weeks of the scanning appointment. Participants were also requested to abstain from alcohol and caffeine consumption, as well as nicotine on the night prior to the scanning day and on the day of scanning. Before the beginning of the MRI session, participants’ urine was biochemically screened with a MULTI 8/2 strip test (Diagnostik Nord, Schwerin, Germany) for the presence of buprenorphine (cutoff 10 ng/mL), amphetamine (cutoff 1000 ng/mL), benzodiazepine (300 ng/mL), cocaine (cutoff 300 ng/mL), methamphetamine (1000 ng/mL), morphine/heroine (cutoff 300 ng/mL), methadone (cutoff 300 ng/mL), THC (cutoff 50 ng/mL). Cutoff levels are those recommended by the American National Institute on Drug Abuse (NIDA^65^). Participants provided informed consent on the use of the urine strip test and agreed to its anonymous data sharing, prior to their participation in the study.

### MRI data acquisition

All magnetic resonance imaging (MRI) data was acquired using a whole-body 3 Tesla scanner (Magnetom Verio, Siemens Healthcare, Erlangen, Germany) equipped with a 32-channel Siemens head coil at the Day Clinic for Cognitive Neurology, University of Leipzig. For all the MRI data provided here, the scanner remained stable and did not undergo any major maintenance or updates that would systematically affect the quality of the acquired data. For each participant the following scans were obtained: 1) a high-resolution structural scan, 2) four rs-fMRI scans, 3) two gradient echo fieldmaps and, 4) two pairs of spin echo images with reversed phase encoding direction. A low-resolution structural image of each participant was acquired using a FLAIR sequence for clinical screening.

#### Structural scan

The high-resolution structural image was acquired using a 3D MP2RAGE sequence^66^ with the following parameters: voxel size=1.0 mm isotropic, FOV=256×240×176 mm, TR=5000 ms, TE=2.92 ms, TI1=700 ms, TI2=2500 ms, flip angle 1=4°, flip angle 2=5°, bandwidth=240 Hz/Px, GRAPPA acceleration with iPAT factor 3 (32 reference lines), pre-scan normalization, duration = 8.22 min. From the two images produced by the MP2RAGE sequence at different inversion times (inv1 and inv2), a quantitative T1 map (t1map), and a uniform T1-weighted image (t1w) were generated. Importantly, the latter image is purely T1-weighted, whereas standard T1-weighted image, for example acquired with the MPRAGE sequence, also contain contributions of proton density and T2^∗^. It should be taken into account that such differences can affect morphometric measures^67^.

For one participant (010025), the structural scan is MPRAGE instead of MP2RAGE (the T1-weighted image file names contain the sequence type) with voxel size=1 mm isotropic, FoV=256×240×176, TR=2300 ms, TE=2.98 ms, TI=900 ms, flip angle=9°, bandwidth=238 Hz/Px.

#### Resting-state scans

Four rs-fMRI scans were acquired in axial orientation using T2^∗^-weighted gradient-echo echo planar imaging (GE-EPI) with multiband acceleration, sensitive to blood oxygen level-dependent (BOLD) contrast^68,69^. Sequences were identical across the four runs, with the exception of alternating slice orientation and phase-encoding direction, to vary the spatial distribution of distortions and signal loss. Thus, the y-axis was aligned parallel to the AC-PC axis for runs 1 and 2, and parallel to orbitofrontal cortex for runs 2 and 4. The phase-encoding direction was A–P for runs 1 and 3, and P–A for runs 2 and 4. Further parameters were set as follows for all four runs: voxel size=2.3 mm isotropic, FOV=202×202 mm^2^, imaging matrix=88×88, 64 slices with 2.3 mm thickness, TR=1400 ms, TE=39.4 ms, flip angle=69°, echo spacing=0.67 ms, bandwidth=1776 Hz/Px, partial fourier 7/8, no pre-scan normalization, multiband acceleration factor=4, 657 volumes, duration=15 min 30 s. During the resting-state scans, participants were instructed to remain awake with their eyes open and to fixate on a crosshair.

#### Scans for distortion correction

Two prominent methods exist to correct for geometric distortions in EPI images: fieldmaps, which represent the degree of distortion as calculated from two phase images with different echo times^70,71^, and reverse phase encoding, in which pairs of “blip-up blip-down” images are acquired with opposite phase encoding direction — thus opposite distortions — and used to model a middle distortion-free image^72,73^. This dataset contains scans required for both methods to accommodate different preprocessing approaches and facilitate method comparison. Before each pair of resting-state runs with the same y-axis orientation (see above), the following scans were acquired in the same orientation as the subsequent resting-state scans: a pair of spin echo images (voxel size=2.3 mm isotropic, FOV=202×202 mm^2^, imaging matrix=88×88, 64 slices with 2.3 mm thickness, TR=2200 ms, TE=52 ms, flip angle=90°, echo spacing=0.67 ms, phase encoding=AP/PA, bandwidth=1776 Hz/Px, partial fourier 6/8, no pre-scan normalization, duration=0.20 min each), and a gradient echo fieldmap (voxel size=2.3 mm isotropic, FOV=202×202 mm^2^, imaging matrix=88×88, 64 slices with 2.3 mm thickness, TR=680 ms, TE1=5.19 ms, TE2=7.65 ms, flip angle=60°, bandwidth=389 Hz/Px, prescan normalization, no partial fourier, duration=2.03 min).

#### Additional scans

109 subjects also took part in a complementary protocol. Therefore, additional modalities will be available for these subjects. Modalities include high-resolution T2-weighted (108 subjects), diffusion-weighted (109), 3D FLAIR (47), phases and magnitudes of gradient-echo images suitable for Susceptibility-Weighted Imaging (SWI), and Quantitative Susceptibility Mapping (QSM) (45 subjects), as well as an additional 15-minute resting-state scan for all 109 subjects.

### MRI data preprocessing

To enhance data usability we provide preprocessed data from 189 subjects (five participants did not have all four resting-state scans available, and were excluded from preprocessing). Data from five participants were further excluded due to failure at the preprocessing stage. The raw MRI data of these subjects are not corrupted, and are therefore available in the main database. Preprocessing pipelines were implemented using Nipype^74^ and are described in more detail below. All code is openly available (https://github.com/NeuroanatomyAndConnectivity/pipelines/tree/master/src/lsd_lemon).

Importantly, the preprocessing performed here is just one out of a multitude of possible pipelines that could be conceived for this dataset. The decisions taken at individual processing steps will not be suitable for every application. Users are strongly advised to familiarize themselves with the details of the workflow before adopting the preprocessed data for their study. We also encourage users to subscribe to the mailing list for updates and discussions regarding the preprocessing pipelines used here (http://groups.google.com/group/resting_state_preprocessing).

#### Structural data

The background of the uniform T1-weighted image was removed using CBS Tools^75^, and the masked image was used for cortical surface reconstruction using FreeSurfer’s full version of recon-all^76,77^. A brain mask was created based on the FreeSurfer segmentation results. Diffeomorphic nonlinear registration as implemented in ANTs SyN algorithm^78^ was used to compute a spatial transformation between the individual’s T1-weighted image and the MNI152 1mm standard space.

To remove identifying information from the structural MRI scans, a mask for defacing was created from the MP2RAGE images using CBS Tools^75^. This mask was subsequently applied to all anatomical scans.

#### Functional data

The first five volumes of each resting-state run were excluded. Transformation parameters for motion correction were obtained by rigid-body realignment to the first volume of the shortened time series using FSL MCFLIRT^79^. The fieldmap images were preprocessed using the fsl_prepare_fieldmap script. A temporal mean image of the realigned time series was rigidly registered to the fieldmap magnitude image using FSL FLIRT^80^ and unwarped using FSL FUGUE^81^ to estimate transformation parameters for distortion correction. The unwarped temporal mean was rigidly coregistered to the subject’s structural scan using FreeSurfer’s boundary-based registration algorithm^82^, yielding transformation parameters for coregistration. The spatial transformations from motion correction, distortion correction, and coregistration were then combined and applied to each volume of the original time series in a single interpolation step. The time series were masked using the brain mask created from the structural image (see above). The six motion parameters and their first derivatives were included as nuisance regressors in a general linear model (GLM), along with regressors representing outliers as identified by Nipype's rapidart algorithm (https://nipype.readthedocs.io/en/latest/interfaces/generated/nipype.algorithms.rapidart.html), as well as linear and quadratic trends. To remove physiological noise from the residual time series, we followed the aCompCor approach as described by Behzadi and colleagues^83^. Masks of the white matter and cerebrospinal fluid were created by applying FSL FAST^84^ to the T1-weighted image, thresholding the resulting probability images at 99%, eroding by one voxel and combining them to a single mask. Of the signal of all voxels included in this mask, the first six principal components were included as additional regressors in a second GLM, run on the residual time series from the first GLM. The denoised time series were temporally filtered to a frequency range between 0.01 and 0.1 Hz using FSL, mean centered and variance normalized using Nitime^85^. The fully preprocessed time series of all for runs were temporally concatenated. To facilitate analysis in standard space, the previously derived transformation was used to project the full-length time series into MNI152 2 mm space. The preprocessed data are made available in the subjects’ native structural space and MNI standard space, along with the subject’s brain mask and all regressors used for denoising.

### Data security and data anonymization procedures

Data for all participants was stored on our instance of the eXtensible Neuroimaging Archive Toolkit (XNAT^86^) v.1.6.5. at the MPI-CBS. Access to the initial project was restricted (via XNAT’s private project mode) to members of the Neuroanatomy & Connectivity Group at MPI-CBS for initial curation and quality assessment of data. All data comprised in the MPI-Leipzig Mind-Brain-Body database were derived from MPI-CBS so data import into XNAT was done from a local secured network.

A specially customized XNAT uploader was used to upload all participants’ data to XNAT. The native DICOM format was used for MRI data, whilst a standard ASCII (^∗^.csv, ^∗^.txt) format was employed to upload all other experimental data such as surveys, test batteries, and demographical data.

The anonymization measures applied to the MRI data consisted of removal of DICOM header tags containing information which could lead to the identification of test subjects as well as the defacing of all structural (NIFTI) scans. Specific surveys and test batteries containing sensitive information are only available via the restricted project in XNAT for which access needs to be applied for (see the Usage Notes section below).

### Code availability

All code that was implemented for data acquisition and processing is available online (https://neuroanatomyandconnectivity.github.io/opendata/). Data handling and computation of summary measures were implemented in Python. The pipeline used for MRI preprocessing is also available (https://github.com/NeuroanatomyAndConnectivity/pipelines/tree/v2.0/src/lsd_lemon, releasev2.0).

The tasks that the participants received were implemented using the Python package PsychoPy2 Experiment Builder v1.81.03^87,88^, OpenSesame 0.27.4^89^, and Presentation® software (Version 16.5, Neurobehavioral Systems, Inc., Berkeley, CA, http://www.neurobs.com). We provide the respective source codes of the Adaptive Visual and Auditory Oddball Target Detection Task e.g.,^55^; cf.^56^ (Oddball; https://github.com/NeuroanatomyAndConnectivity/opendata/tree/master/scripts), Conjunctive Continuous Performance Task^51^ (CCPT; https://github.com/NeuroanatomyAndConnectivity/ConjunctiveContinuousPerformanceTask), and Emotional Task Switching^50,58^ (ETS; https://github.com/NeuroanatomyAndConnectivity/opendata/tree/master/scripts).

## Data Records

### Survey and task data

Data from all questionnaires are open access, except for two (Facebook Intensity Scale^28^ and Multi-Gender Identity Questionnaire^35^). Results of questionnaires are released as summary scores, except for: Multi-Gender Identity Questionnaire^35^ (MGIQ), Mobile Phone Usage (MPU), Facebook Intensity Scale^28^ (FBI), New York Cognition Questionnaire^49^ (NYC-Q), and the short version of the New York Cognition Questionnaire^9^ (Short-NYC-Q). Task data for the CCPT^51^, ETS^50,58^, and oddball task e.g.,^55^; cf.^56^ are available via subject-specific .csv files. Accompanying specifications and information for each questionnaire and task are given in .txt file format.

A basic demographic summary is provided together with general information on data acquisition. The metafile includes gender, age (5-year bins), body mass index, handedness, current or past diagnosed psychiatric disorder(s), result of the drug test on day of scanning, and formal education.

Behavioral and questionnaire data is provided at https://dataverse.harvard.edu/ through the following link https://doi.org/10.7910/DVN/VMJ6NV (Data Citation 2).

### MRI data

The dataset is organized in concordance with the Brain Imaging Data Structure (BIDS) format^90^. This facilitates data analysis, for example with BIDS-Apps^91^ (http://bids-apps.neuroimaging.io). BIDS-Apps encapsulate standard MRI analysis tools within an application that understands the BIDS format and allows to automatically access relevant data and metadata.

MRI data are currently available from three locations:

1. OpenfMRI.org (now renamed to OpenNeuro) platform also hosts the raw data (Data Citation 3)
2. The OpenfMRI repository provides API access available via https://openfmri.org/dataset/api/. In addition, similar to all other datasets in OpenfMRI, our dataset is available via Amazon Web Services S3 object data store under the s3://openneuro/ds000221/
3. International Neuroimaging Data-sharing Initiative, INDI (Data Citation 4)
4. Gesellschaft für wissenschaftliche Datenverarbeitung mbH Göttingen (GWDG): https://www.gwdg.de/
5. Raw and preprocessed data at this location is accessible through web browser (https://ftp.gwdg.de/pub/misc/MPI-Leipzig_Mind-Brain-Body/) and a fast FTP connection (ftp://ftp.gwdg.de/pub/misc/MPI-Leipzig_Mind-Brain-Body/). In the case the location of the data changes in the future, the location of the dataset can be resolved with PID 21.11101/0000-0004-2CD6-A (e.g., https://hdl.handle.net/21.11101/0000-0004-2CD6-A).

## Technical Validation

All datasets were manually assessed for missing or corrupt data (see Supplementary Table 3 and Supplementary File 1). Further quality control of the data was applied to the MRI and behavioral measures, as described below.

### MRI data quality assessment

Preprocessed MRI data were assessed for quality using the mriqc package^92^ (the code was adapted from https://github.com/chrisfilo/mriqc and can be found at https://github.com/NeuroanatomyAndConnectivity/pipelines/tree/master/src/lsd_lemon, release v2.0), implemented in Python. mriqc creates a report for each individual scan based on assessment of movement parameters, coregistration, and temporal signal-to-noise (tSNR) calculations. For comparison, all individual-level scores are displayed with respect to the group-level distribution. We visually inspected the quality assessment reports for each subject to ensure adequate coregistration and fieldmap correction.

**Figure 2:**
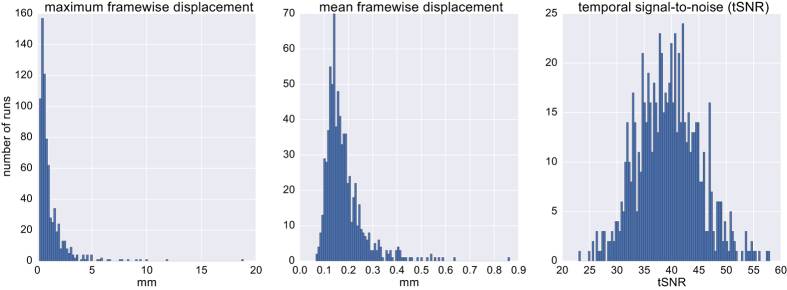
Quality assessment of resting-state fMRI scans. Distribution of motion (maximum and mean framewise displacement).

As motion during the resting-state fMRI scan poses a substantial source of noise^93^, we characterized motion for each run as the mean and maximum framewise displacement (Fig. 2). Overall, the summary of motion parameters demonstrates that the data are largely of sufficient quality, with 89.2% of runs showing less than one voxel (2.3 mm) maximum framewise displacement, and a mean framewise displacement of 0.18 mm (SD=0.08 mm).

**Figure 3:**
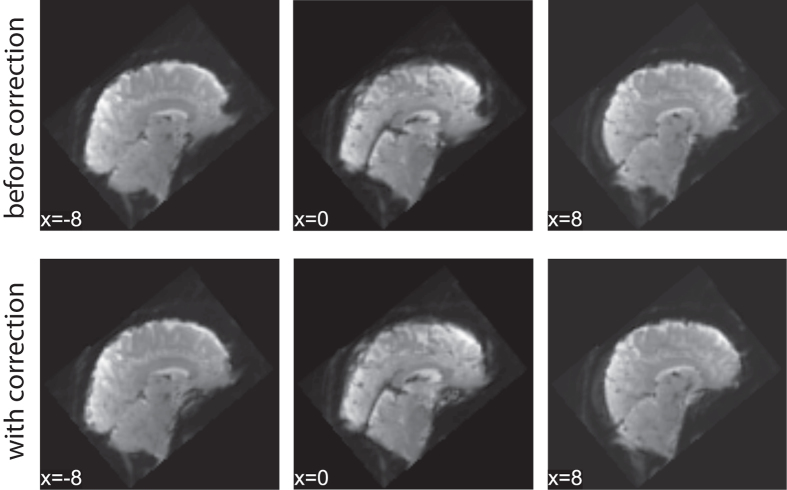
Example impact of fieldmap correction.

Fieldmap correction provides an approach to correct for distortions due to susceptibility artifacts. While unable to recover signal loss, the correction of such nonlinear distortions improves coregistration between scan types, and group-level alignment^94^. As an example, we present a single dataset, pre- and post-fieldmap correction, in Fig. 3. As expected, fieldmap correction primarily shifted voxels within ventral regions.

**Figure 4:**
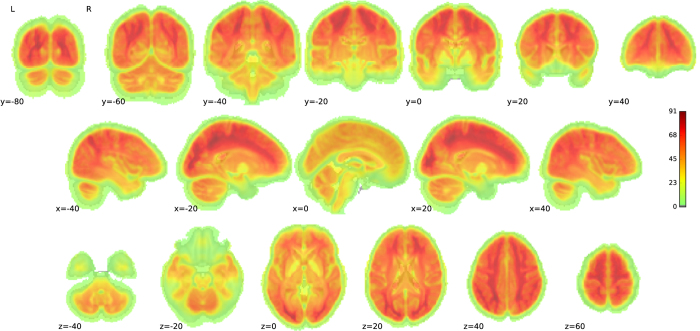
Temporal signal-to-noise (tSNR). Group-level variance in temporal signal-to-noise (tSNR) across the brain. tSNR values are lower in ventral regions including orbitofrontal and temporal cortex.

Temporal signal-to-noise (tSNR), which is calculated on the voxel-level as the mean signal divided by the standard deviation, offers a general overview of the local differences across the brain. We observed lower tSNR in ventral regions, including the orbitofrontal and temporal cortex (Fig. 4).

### Behavioral measures quality assessment

Fifteen questionnaires without a published German version were in-house translated (English-German). To ensure general usability of the translated questionnaires, their reliability was estimated using Cronbach’s Alpha coefficient (see Table 3). For comparison, the Cronbach’s Alpha coefficients from the original questionnaires are also reported in Table 3.

Internal consistency^95^ of the majority of questionnaires was acceptable, with an average Cronbach’s Alpha of 0.78, thus showing that the German translations of those specific questionnaires are reproducible and valid. However, three questionnaires (Short Dark Triad^40^, Body Consciousness Questionnaire^22^, and the Creative Achievement Questionnaire^61^) and four scales (two scales of the Five Facets of Mindfulness Questionnaire^46^, one scale of the Metacognition Questionnaire^47^, and one scale of the Involuntary Musical Imagery Scale^34^) showed modest reliability, with Cronbach’s Alpha coefficient &lt;0.70, and should be interpreted with caution.

## Usage Notes

The MRI dataset can be accessed at https://openneuro.org, http://fcon_1000.projects.nitrc.org, or https://www.gwdg.de/ and the behavioral data is available at http://www.nitrc.org (http://nitrc.org/projects/mpilmbb/). The following data are publicly available: 1) MRI data (structural and functional), 2) general demographic of the studied population, 3) summary scores and/or indexes of the questionnaires and tasks, and 4) raw scores of the measures that do not possess summary scores and have not been classified as sensitive. All MRI datasets are made available in NIFTI format, and all anatomical scans have been defaced.

The dataset, protocols, and software used in the acquisition and processing of the data are documented, curated, and available for download. For access to the behavioral data, users must first agree to the terms of data usage, which prohibit any usage that aims to identify the individuals based on these phenotypic data.

### Additional access to sensitive behavioral measures

Individual behavioral scores and sensitive phenotypic measures may be made available upon request to the corresponding authors. The completion of additional data license and confidentiality forms will be required in advance of further data access.

## Additional information

**How to cite this article**: Mendes, N. *et al*. A functional connectome phenotyping dataset including cognitive state and personality measures. *Sci. Data*. 6:180307 https://doi.org/10.1038/sdata.2018.307 (2019).

**Publisher’s note**: Springer Nature remains neutral with regard to jurisdictional claims in published maps and institutional affiliations.

## Supplementary Material



Supplementary File 1

Supplementary Table 1

Supplementary Table 2

Supplementary Table 3

## Figures and Tables

**Table 1:** Phases of the data acquisition.

Day 1 (MPI CBS)	Day 2 (Home)	Day 3 (Uni Clinic)	Day 4 (Home)	Day 5 (MPI CBS)
ASR	ACS	Scanning session	AMAS	BIS/BAS
God-MSI	BDI		NEO PI-R	CAQ
IAT	BP	FBI		MCQ-30
IMIS	ESS	S-D=MW		BCQ
MGIQ	HADS	Short-NYC-Q_inscan1-4		FFMQ
SCS	MMI	Short-NYC-Q_postETS		RAT
SD3	MPU	NYC-Q_postscan		SYN
SE	PSSI	ETS		AUT
TPS		CCPT		TCIA
VISQ		Oddball		
UPPS-P				
SDS				
Overview of the different phases of the data acquisition. **Note. MPI-CBS**=Max Planck Institute for Human Cognitive and Brain Sciences, Leipzig; **Uni Clinic**= Day Clinic for Cognitive Neurology, University of Leipzig. **ACS**=Attention Control Scale, **AMAS**=Abbreviated Math Anxiety Scale, **ASR**=Adult Self Report, **AUT**=Alternative Uses Task, **BCQ**=Body Consciousness Questionnaire, **BDI**=Beck Depression Inventar-II, **BIS/BAS**=Behavioral Inhibition and Approach System, **BP**=Boredom Proneness Scale, **CAQ**=Creative Achievement Questionnaire, **CCPT**=Conjunctive Continuous Performance Task, **ESS**=Epworth Sleepiness Scale, **ETS**=Emotional task switching; **FBI**=Facebook Intensity Scale, **FFMQ**=Five Facets of Mindfulness Questionnaire, **Gold-MSI**=Goldsmiths Musical Sophistication Index, **HADS**=Hospital Anxiety and Depression Scale, **IAT**=Internet Addiction Test, **IMIS**=Involuntary Musical Imagery Scale, **MCQ-30**=Metacognition Questionnaire, **MGIQ**=Multi-Gender Identity Questionnaire, **MMI**=Multimedia Multitasking Index, **MPU**=Mobile Phone Usage, **NEO PI-R**=NEO Personality Inventory-Revised, **NYC-Q_postscan**=New York Cognition Questionnaire after scan, **Oddbal**l=Adaptive Visual and Auditory Oddball Target Detection Task, **PSSI**=Personality Style and Disorder Inventory, **RAT**=Remote Associates Test, **SCS**=Brief Self-Control Scale, **SD3**=Short Dark Triad, **S-D-MW**=Spontaneous and Deliberate Mind-Wandering, **SDS**=Social Desirability Scale-17; **SE**=Self-Esteem Scale, **Short-NYC-Q_inscan1-4**=Short Version of the New York Cognition Questionnaire in scanner, **Short-NYC-Q_postETS**=Short Version of the New York Cognition Questionnaire after tasks, **SYN**=Synesthesia Color Picker Test, **TCIA**=Test of Creative Imagery Abilities, **TPS**=Tuckman Procrastination Scale, **UPPS-P**=UPPS-P Impulsive Behavior Scale, **VISQ**=Varieties of Inner Speech Questionnaire.				

**Table 2:** Behavioral measures.

Abbreviation	Behavioral Measure	N
***Personality and Habitual Behaviors***		
AMAS	Abbreviated Math Anxiety Scale^9^	145
ASR	Adult Self Report adapted from^13^	213
BDI	Beck Depression Inventar-II^14–16^	210
BIS/BAS	Behavioral Inhibition and Approach System^17–20^	288
BCQ	Body Consciousness Questionnaire^21^	79
BP	Boredom Proneness Scale^22^	209
SCS	Brief Self-Control Scale^23,24^	214
ESS	Epworth Sleepiness Scale^25,26^	210
FBI	Facebook Intensity Scale^27^	180
Gold-MSI	Goldsmiths Musical Sophistication Index^28,29^	214
HADS	Hospital Anxiety and Depression Scale^30,31^	210
IAT	Internet Addiction Test^32^	214
IMIS	Involuntary Musical Imagery Scale^33^	214
MPU	Mobile Phone Usage	210
MGIQ	Multi-Gender Identity Questionnaire^34^	159
MMI	Multimedia Multitasking Index^35^	209
NEO PI-R	NEO Personality Inventory-Revised^10–12^	169
PSSI	Personality Style and Disorder Inventory^36^	209
SE	Self-Esteem Scale^38^	214
SD3	Short Dark Triad^39^	213
SDS	Social Desirability Scale-17^40,96^	214
TPS	Tuckman Procrastination Scale^41^	214
UPPS-P	UPPS-P Impulsive Behavior Scale^42,43^, cf.^44^	214
***Mind-Wandering and Mindfulness***		
FFMQ	Five Facets of Mindfulness Questionnaire^45^	79
MCQ-30	Metacognition Questionnaire^46,47^	79
NYC-Q_posttasks	New York Cognition Questionnaire^48^	202
NYC-Q_postscan		188
NYC-Q		
Short-NYC-Q_inscan1	Short Version of the New York Cognition Questionnaire^8^	175
Short-NYC-Q_inscan2		174
Short-NYC-Q_inscan3		174
Short-NYC-Q_inscan4		170
Short-NYC-Q_postETS		181
Short-NYC-Q_prescan		159
S-D-MW	Spontaneous and Deliberate Mind-Wandering^51^; cf.^44^	214
VISQ	Varieties of Inner Speech Questionnaire^52^	214
***Synesthesia***		
SYN	Synesthesia Color Picker Test (synesthete.org)^53^	73
***Cognitive Control and Sustained Attention***		
Oddball	Adaptive Visual and Auditory Oddball Target Detection Task e.g.,^54^, cf.^55^	137
ACS	Attention Control Scale^56^	210
CCPT	Conjunctive Continuous Performance Task^50^	169
ETS	Emotional Task Switching^49^; see^57^	189
***Creativity***		
AUT	Alternative Uses Task^58,59^	77
CAQ	Creative Achievement Questionnaire^60^	79
RAT	Remote Associates Test cf.^61,^^62^	77
TCIA	Test of Creative Imagery Abilities^63^	77
Overview of data available for each questionnaire and task.		

**Table 3:** Reliability of translated questionnaires.

Translated Questionnaires	Cronbach’s alpha coefficient (α)
***Personality and Habitual Behaviors***	
Abbreviated Math Anxiety Scale^9^	α=0.92 (English original: α=0.90)
Body Consciousness Questionnaire^21^	*Private body scale*, α=0.63*Public body scale*, α=0.62 *Body competence scale*, α=0.62Note. Cronbach’s α coefficients of the original scales are not available
Boredom Proneness Scale^22^	α=0.84 (English original: α=0.79)
Internet Addiction Test^32^	α=0.91 (item 3 was excluded from the analysis due to different scaling; values for English unavailable)
Involuntary Musical Imagery Scale^33^	*Negative valence*, α=0.88 for (English original: α=0.91)*Movement*, α=0.92 (English original: α=0.88)*Personal reflections*, α=0.64 (English original: α=0.76)*Help*, α=0.90 (English original: α=0.84)
Multimedia Multitasking Index^35^	α=0.97 (English original: not reported)
Self-Esteem Scale^38^	α=0.88 (English original: α=0.79 for males; α=0.83 for females)
Short Dark Triad^39^	*Machiavellianism*, α=0.68 (English original: α=0.78)*Narcissism*, α=0.65 (English original: α=0.77)*Psychopathy*, α=0.59 for (English original: α=0.80)
UPPS-P Impulsive Behavior Scale^42,^^43^, cf.^45^	*Negative urgency*, α=0.83 (English original: α=0.90)*Lack of premeditation*, α=0.75 (English original: α=0.91)*Lack of perseverance*, α=0.84 (English original: α=0.82)*Sensation seeking*, α=0.82 (English original: α=0.86)*Positive urgency*, α=0.90 (English original: not reported)
***Mind-Wandering and Mindfulness***	
Five Facets of Mindfulness^46^	*Observing scale,* α=0.68 (English original: α=0.83)*Describing scale*, α=0.89 (English original: α=0.91)*Acting with Awareness scale,* α=0.70 (English original: α=0.87)*Nonjudging scale*, α=0.87 (English original: α=0.87)*Nonreactivity scale,* α=0.69 (English original: α=0.75)
Metacognition Questionnaire^46,^^48^	*Cognitive confidence,* α=0.80 (English original: α= 0.93)*Positive beliefs,* α=0.85 (English original: α= 0.92)*Cognitive self-consciousness,* α=0.85 (English original: α=0.92)*Uncontrollability and danger,* α=0.80 (English original: α= 0.91)*Need to control thoughts,* α=0.67 (English original: α=0.72)
Spontaneous and Deliberate Mind-Wandering^51,^ cf.^45^	*Deliberate mind-wandering*, α=0.81 (English original: α=0.90)*Spontaneous mind-wandering*, α=0.81 (English original: α=0.88)
Varieties of Inner Speech Questionnaire^52^	*Dialogic inner speech*, α=0.74 (English original: α=0.83)*Condensed inner speech*, α=0.79 (English original: α=0.83)*Other people in inner speech*, α=0.86 (English original: α=0.88)*Evaluative inner speech*, α=0.74 (English original: α=0.80)
***Cognitive Control and Sustained Attention***	
Attention Control Scale^56^	α=0.74 (English original: not reported)
***Creativity***	
Creative Achievement Questionnaire^60^	α=0.67 (English original: α=0.96)
Estimated reliability of the English-German translated questionnaires using Cronbach’s Alpha coefficient (α).	
**Note**. Cronbach’s alpha coefficient was not computed for the NYC-Q and the Short-NYC-Q, as the heterogeneity of items within these questionnaires do not describe a unitary phenomenon and are not designed to be internally consistent^94^. We recommend a factor analytic approach to derive behavioral scores from these questionnaires (see^48^).	

## Exclusion criteria. Exclusion criteria to prospective participants.

**Exclusion Criteria**

- History of psychiatric diseases that required inpatient treatment for longer than 2 weeks within the last 10 years (e.g., psychosis, attempted suicide, post-traumatic stress disorder);
- History of neurological disorders (incl. multiple sclerosis, stroke, epilepsy, brain tumour, meningoencephalitis, severe concussion);
- History of malignant diseases;
- Intake of one of the following medications:
- Any centrally active drugs (including *Hypericum perforatum*)
- Beta- and alpha-blocker
- Cortisol
- Any chemotherapeutic or psychopharmacological medication;
- Positive drug anamnesis (extensive alcohol, MDMA, amphetamines, cocaine, opiates, benzodiazepine, cannabis);
- Extensive testing experience at the MPI-CBS or other academic institution;
- Past or present student of Psychology;
- MRI exclusion criteria (see Supplementary Table 2)
- Any metallic implants, braces, non-removable piercings
- Tattoos
- Pregnancy
- Claustrophobia
- Tinnitus
- Surgical operation in the last 3 months
