# Supplementary material for: A functional connectome phenotyping dataset including cognitive state and personality measures
Source: Sci Data. 2019 Feb 12;6:180307. doi: 10.1038/sdata.2018.307 (PMC6371896; doi:10.1038/sdata.2018.307)
Supplement: Supplementary File 1 [file sdata2018307-s2.docx]

**Description of codes used in Supplementary Tables 1 and 3**

**BPS: Boredom Proneness Scale**

BPS_sum = sum score

**TAP_WM: TAP working memory**

TAP_WM_1 = mean reaction time

﻿TAP_WM_2 = median reaction time

﻿TAP_WM_3 = percent rank of the reaction time (compared to norm sample)

﻿TAP_WM_4 = standard deviation

﻿TAP_WM_5 = percent rank of the standard deviation (compared to norm sample)

﻿TAP_WM_6 = how many correct matches

﻿TAP_WM_7 = how many incorrect matches (errors)

﻿TAP_WM_8 = percent rank of the correct answers (compared to norm sample)

﻿TAP_WM_9 = how many missed matches (omissions)

﻿TAP_WM_10 = percent rank of the omissions (compared to norm sample)

﻿TAP_WM_11 = outliers

**WST: Vocabulary test**

WST_1 = WST raw data; how many real words did the participant correctly indicated

WST_2 = z-scale (Mean = 0, SD = 1)

WST_3 = IQ-scale (Mean = 100, SD = 15)

WST_4 = Z-scale (Mean = 100, SD = 10)

**SCS: Brief Self-Control Scale**

SCS_SelfCtrl_sum: self-control sum score

**SD3:** **The Short Dark Triad**

SD3_Mach_sum = machiavellianism sum score

SD3_Narc_sum = narcissism sum score

SD3_Psycho_sum = psychopathy sum score

**NYCQ: New York Cognition Questionnaire**

NYCQ_post-ses-01_1 till NYCQ_post-ses-01_31 = NYCQ items measured after first scanning session (after first MRI acquisition)

NYCQ_post-ses-02_1 till NYCQ_post-ses-02_31 = NYCQ items measured after second scanning session (after second MRI acquisition at day 3)

NYCQ_post-tasks_1 till NYCQ_post-tasks_23 = The first 23 NYCQ items measured after task block including emotional task switching and continuous performance task.

**TCIA: Test of Creative Imagery Abilities**

TCIA_Vividness_mean = vividness mean score

TCIA_Orig_mean = originality mean score

TCIA_Transform_mean = transformativeness mean score

**HADS: Hospital Anxiety and Depression Scale**

HADS-A_summary_sum = anxiety sum scores

HADS-D_summary_sum = depression sum scores

**IMIS: Involuntary Musical Imagery Scale**

IMIS_NegVal_sum = negative valence sum scores (subjective evaluation)

IMIS_Help_sum = help sum scores (beneficial and constructive aspects)

IMIS_Movement_sum = movement sum scores (embodied responses)

IMIS_PersRef_sum = personal reflections sum scores (personal qualities)

**LPS: Logical Deductive Thinking Questionnaire**

LPS_1 = LPS raw data, how many symbol-rows did the participant process correctly

**CAQ: Creative Achievement Questionnaire**

CAQ_score = cumulated creative achievement scores

**ASR: Adult self-report (18-59)**

ASR_summary_adaptiveFunctioning_friends_sum = adaptive functioning (friends subscore)

ASR_summary_adaptiveFunctioning_spouse_sum = adaptive functioning (spouse subscore)

ASR_summary_adaptiveFunctioning_family_sum = adaptive functioning (familiy subscore)

ASR_summary_adaptiveFunctioning_job_sum = adaptive functioning (job subscore)

ASR_summary_adaptiveFunctioning_education_sum = adaptive functioning (education subscore)

ASR_scale_substanceUse_tabaco_perday = tobacco usage per day (average in the last 6 months)

ASR_scale_substanceUse_alcohol_daysdrunk = days being drunk (amount in the last 6 months)

ASR_scale_substanceUse_drugs_daysused = days being drugged (amount in the last 6 months)

ASR_summary_criticalItems_sum = amount of individual problems

ASR_summary_syndromeProfiles_anxiousdepressed_sum = anxoius-depressed syndrome scale sum

ASR_summary_syndromeProfiles_withdrawn_sum = withdrawn syndrome scale sum

ASR_summary_syndromeProfiles_somaticComplaints_sum = somatic complaints syndrome scale sum

ASR_summary_syndromeProfiles_thoughtProblems_sum =thought problems syndrome scale sum

ASR_summary_syndromeProfiles_attentionProblems_sum = attention problems syndrome scale sum

ASR_summary_syndromeProfiles_aggressiveBehavior_sum = aggressive behavior syndrome scale sum

ASR_summary_syndromeProfiles_rulebreakingBehavior_sum = rule breaking behavior syndrome scale sum

ASR_summary_syndromeProfiles_intrusive_sum = intrusive behavior syndrome scale sum

ASR_summary_syndromeProfiles_internalizing_sum = internalizing behavior scale sum

ASR_summary_syndromeProfiles_externalizing_sum = externalizing behavior scale sum

**MMI: Multimedia Multitasking Index**

MMI_score = index of media multitasking intensity

**NEO: NEO-Personality Inventory – Self-Description-Form**

NEO_N = neuroticism sum score

NEO_N1_anx = neuroticism facet anxiety sum score

NEO_N2_host = neuroticism facet hostility sum score

NEO_N3_depr = neuroticism facet depression sum score

NEO_N4_selfcon = neuroticism facet self-consciousness sum score

NEO_N5_imp = neuroticism facet impulsivity sum score

NEO_N6_vuln = neuroticism facet vulnerability sum score

NEO_E = extraversion sum score

NEO_E1_warm = extraversion facet warmth sum score

NEO_E2_greg = extraversion facet gregariousness sum score

NEO_E3_ass = extraversion facet assertiveness sum score

NEO_E4_act = extraversion facet activity sum score

NEO_E5_excseek = extraversion facet excitement seeking sum score

NEO_E6_PosEmo = extraversion facet positive emotions sum score

NEO_O = openness to experience sum score

NEO_O1_fan = openness facet fantasy sum score

NEO_O2_aest = openness facet aesthetics sum score

NEO_O3_feel = openness facet feelings sum score

NEO_O4_act = openness facet actions sum score

NEO_O5_idea = openness facet ideas sum score

NEO_O6_value = openness facet values sum score

NEO_A = agreeableness sum score

NEO_A1_trust = agreeableness facet trust sum score

NEO_A2_sf = agreeableness facet straightforwardness sum score

NEO_A3_altr = agreeableness facet altruism sum score

NEO_A4_compl = agreeableness facet compliance sum score

NEO_A5_modes = agreeableness facet modesty sum score

NEO_A6_tenmind = agreeableness facet tendermindedness sum score

NEO_C = conscientiousness sum score

NEO_C1_comp = conscientiousness facet competence sum score

NEO_C2_order = conscientiousness facet order sum score

NEO_C3_dutif = conscientiousness facet dutifulness sum score

NEO_C4_achstr = conscientiousness facet achievement striving sum score

NEO_C5_selfdis = conscientiousness facet self-discipline sum score

NEO_C6_deli = = conscientiousness facet deliberation sum score

**ACS: Attention Control Scale**

ACS_sum = attention control sum score

**VIS: Varieties of Inner Speech Questionnaire**

VIS_dialog_sum = dialogic inner speech sum score

VIS_condensed_sum = condensed inner speech sum score

VIS_other_sum = voice of others in the inner speech sum score

VIS_eval_sum = evaluative/motivational inner speech sum score

**ESS: Epworth Sleepiness Scale**

ESS_summary_sum = sleepiness sum score

**MPU: Mobile phone usage**

MPU_1. Do you own a cell phone?

0= no; 1= yes

MPU_2. How often do you carry you cell phone with you?

A1= the whole day; A2= most time of the day; A3= a couple of hours a day

MPU_3. How many text messages do you send a week (on average)?

MPU_4. How many phone calls (using your mobile phone) do you make/take per week (on average)?

MPU_5. Do you own a smartphone (iPhone, Android etc. - a multipurpose phone with internet connection)?

0= no; 1= yes

MPU_6. Do you have a flat internet rate?

MPU_7. How long have you been using a smartphone?

(months, years)

MPU_8. Do you use your smartphone to browse the web?

If yes, how often do you look up facts on your phone in a social situations?

A1= never; A2= sometimes; A3= often

MPU_9. Do you use your smartphone to check your emails?

If yes, do you get a notification each time you get a new email or do you have to check it manually?

MPU_10. If manually, how often?

A1= never; A2= sometimes; A3= often

MPU_11. Do you use your phone for social networking (Facebook, Twitter, Google+ etc.)?

A1= never; A2= sometimes; A3= often

MPU_12. Do you post messages (including tweets) to social networks using your phone (as opposed to just reading)?

MPU_13. Do you use your phone instant messaging (Viber, WhatsApp, Google chat, Facebook messaging, etc.)?

A1= never; A2= sometimes; A3= often

MPU_14. Do you use you phone to check the news?

A1= never; A2= sometimes; A3= often

MPU_15. Do you use your phone to take and share pictures?

A1= never; A2= sometimes; A3= often

MPU_16. In a social situation such as a dinner with friends, how often do you have an urge to check your phone (to check the news, Facebook, etc.)?

A1= never; A2= sometimes; A3= often

MPU_17. How often do you resist this urge due to a social pressure?

A1= never; A2= sometimes; A3= often

MPU_18. Do you read printed newspapers/magazines?

A1= never; A2= sometimes; A3= often

MPU_19. How many books do you read a year?

**RWT - Regensburger Wortflüssigkeits-Test: fluidness of ideas & cognitive flexibility**

*S-words*

RWT_1 = how many s-words did the participant name during the first minute (according to rules)

RWT_2 = percentile rank of correct words for the subtest 's-words' (1 minute)

RWT_3 = how many repetitions during the first minute

RWT_4 = how many rule breaks during the first minute

RWT_5 = how many s-words did the participant name during the second minute (according to rules)

RWT_6 = how many repetitions during the second minute

RWT_7 = how many rule breaks during the second minute

RWT_8 = how many s-words did the pp. name in total (according to rules); two minutes

RWT_9 = percentile rank of correct words for the subtest 's-words' (2 minutes)

RWT_10 = how many repetitions in total; two minutes

RWT_11 = how many rule breaks in total; two minutes

*Animals*

RWT_13 = how many animals did the participant. name in the first minute (according to rules)

RWT_14 = percentile rank of correct words for the subtest 'animal' (1 minute)

RWT_15 = how many repetitions during the first minute

RWT_16 = how many rule breaks during the first minute

RWT_17 = how many animals did the pp. name during the second minute (according to rules)

RWT_18 = how many repetitions during the second minute

RWT_19 = how many rule breaks during the second minute

RWT_20 = how many animal-words did the pp. name in total (according to rules); two minutes

RWT_21 = percentile rank of correct words for the subtest 'animal' (2 minutes)

RWT_22 = how many repetitions in total; two minutes

RWT_23 = how many rule breaks in total; two minutes

**BISBAS: Behavioral Inhibition and Approach System**

BISBAS_BIS_sum = behavioral inhibition sum score

BISBAS_BAS_sum = behavioral activation sum score

**SNYCQ: Short version of the NYCQ**

*file name description:*

pres-ses = pre scanning measurement

post-ses = post scanning measurement

run-01 = indicates measurement after resting state scan 1 or 2

run-02 =indicates measurement after resting state scan 3 or 4

acq-AP = indicates measurement after a scan with an anterior-to-posterior acquisition (the first in a run of two scans)

acq-PA = indicates measurement after a scan with a posterior-to-anterior acquisition (the second in a run of two scans)

task-ETS = emotional task switching

task-CPTS = continuous performance task

positive = positive thoughts

negative = negative thoughts

future = future thoughts

past = past thoughts

myself = thoughts about self

people = thoughts about other people

surrpundings = thoughts about surrounding environment

vigilance = self-assessed vigilance

images = thoughts in the form of images

words = thoughts in the form of words

vague = thoughts rather specific or vague

intrusive = intrusiveness of the thoughts

SNYCQ_pre-ses-02_positive = positive thoughts

SNYCQ_pre-ses-02_negative =negative thoughts

SNYCQ_pre-ses-02_future = future thoughts

SNYCQ_pre-ses-02_past = past thoughts

SNYCQ_pre-ses-02_myself = thoughts about self

SNYCQ_pre-ses-02_people = thoughts about other people

SNYCQ_pre-ses-02_surroundings = thoughts about surrounding environment

SNYCQ_pre-ses-02_vigilance = self-assessed vigilance

SNYCQ_pre-ses-02_images = thoughts in the form of images

SNYCQ_pre-ses-02_words = thoughts in the form of words

SNYCQ_pre-ses-02_vague = thoughts rather specific or vague

SNYCQ_pre-ses-02_intrusive = intrusiveness of the thoughts

SNYCQ_post-ses-02-run-01-acq-AP_positive = positive thoughts

SNYCQ_post-ses-02-run-01-acq-AP_negative =negative thoughts

SNYCQ_post-ses-02-run-01-acq-AP_future = future thoughts

SNYCQ_post-ses-02-run-01-acq-AP_past = past thoughts

SNYCQ_post-ses-02-run-01-acq-AP_myself = thoughts about self

SNYCQ_post-ses-02-run-01-acq-AP_people = thoughts about other people

SNYCQ_post-ses-02-run-01-acq-AP_surroundings = thoughts about surrounding environment

SNYCQ_post-ses-02-run-01-acq-AP_vigilance = self-assessed vigilance

SNYCQ_post-ses-02-run-01-acq-AP_images = thoughts in the form of images

SNYCQ_post-ses-02-run-01-acq-AP_words = thoughts in the form of words

SNYCQ_post-ses-02-run-01-acq-AP_specific = thoughts rather specific or vague

SNYCQ_post-ses-02-run-01-acq-AP_intrusive = intrusiveness of the thoughts

SNYCQ_post-ses-02-run-01-acq-PA_positive = positive thoughts

SNYCQ_post-ses-02-run-01-acq-PA_negative = negative thoughts

SNYCQ_post-ses-02-run-01-acq-PA_future = future thoughts

SNYCQ_post-ses-02-run-01-acq-PA_past = past thoughts

SNYCQ_post-ses-02-run-01-acq-PA_myself = thoughts about self

SNYCQ_post-ses-02-run-01-acq-PA_people = thoughts about other people

SNYCQ_post-ses-02-run-01-acq-PA_surroundings = thoughts about surrounding environment

SNYCQ_post-ses-02-run-01-acq-PA_vigilance = self-assessed vigilance

SNYCQ_post-ses-02-run-01-acq-PA_images = thoughts in the form of images

SNYCQ_post-ses-02-run-01-acq-PA_words = thoughts in the form of words

SNYCQ_post-ses-02-run-01-acq-PA_specific = thoughts rather specific or vague

SNYCQ_post-ses-02-run-01-acq-PA_intrusive = intrusiveness of the thoughts

SNYCQ_post-ses-02-run-02-acq-AP_positive = positive thoughts

SNYCQ_post-ses-02-run-02-acq-AP_negative = negative thoughts

SNYCQ_post-ses-02-run-02-acq-AP_future = future thoughts

SNYCQ_post-ses-02-run-02-acq-AP_past = past thoughts

SNYCQ_post-ses-02-run-02-acq-AP_myself = thoughts about self

SNYCQ_post-ses-02-run-02-acq-AP_people = thoughts about other people

SNYCQ_post-ses-02-run-02-acq-AP_surroundings = thoughts about surrounding environment

SNYCQ_post-ses-02-run-02-acq-AP_vigilance = self-assessed vigilance

SNYCQ_post-ses-02-run-02-acq-AP_image = thoughts in the form of images

SNYCQ_post-ses-02-run-02-acq-AP_words = thoughts in the form of words

SNYCQ_post-ses-02-run-02-acq-AP_specific = thoughts rather specific or vague

SNYCQ_post-ses-02-run-02-acq-AP_intrusive = intrusiveness of the thoughts

SNYCQ_post-ses-02-run-02-acq-PA_positive = positive thoughts

SNYCQ_post-ses-02-run-02-acq-PA_negative = negative thoughts

SNYCQ_post-ses-02-run-02-acq-PA_future = future thoughts

SNYCQ_post-ses-02-run-02-acq-PA_past = past thoughts

SNYCQ_post-ses-02-run-02-acq-PA_myself = thoughts about self

SNYCQ_post-ses-02-run-02-acq-PA_people = thoughts about other people

SNYCQ_post-ses-02-run-02-acq-PA_surroundings = thoughts about surrounding environment

SNYCQ_post-ses-02-run-02-acq-PA_vigilance = self-assessed vigilance

SNYCQ_post-ses-02-run-02-acq-PA_images = thoughts in the form of images

SNYCQ_post-ses-02-run-02-acq-PA_words = thoughts in the form of words

SNYCQ_post-ses-02-run-02-acq-PA_specific = thoughts rather specific or vague

SNYCQ_post-ses-02-run-02-acq-PA_intrusive = intrusiveness of the thoughts

SNYCQ_post-ses-02-task-ETS_positive = positive thoughts

SNYCQ_post-ses-02-task-ETS_negative = negative thoughts

SNYCQ_post-ses-02-task-ETS_future = future thoughts

SNYCQ_post-ses-02-task-ETS_past = past thoughts

SNYCQ_post-ses-02-task-ETS_myself = thoughts about self

SNYCQ_post-ses-02-task-ETS_people = thoughts about other people

SNYCQ_post-ses-02-task-ETS_surroundings = thoughts about surrounding environment

SNYCQ_post-ses-02-task-ETS_vigilance = self-assessed vigilance

SNYCQ_post-ses-02-task-ETS_images = thoughts in the form of images

SNYCQ_post-ses-02-task-ETS_words = thoughts in the form of words

SNYCQ_post-ses-02-task-ETS_vague = thoughts rather specific or vague

SNYCQ_post-ses-02-task-ETS_intrusive = intrusiveness of the thoughts

Participant_id.1 = WHAT IS THIS?

SNYCQ_post-ses-02-task-CPTS_positive = positive thoughts

SNYCQ_post-ses-02-task-CPTS_negative = negative thoughts

SNYCQ_post-ses-02-task-CPTS_future = future thoughts

SNYCQ_post-ses-02-task-CPTS_past = past thoughts

SNYCQ_post-ses-02-task-CPTS_myself = thoughts about self

SNYCQ_post-ses-02-task-CPTS_people = thoughts about self

SNYCQ_post-ses-02-task-CPTS_surroundings = thoughts about surrounding environment

SNYCQ_post-ses-02-task-CPTS_vigilance = self-assessed vigilance

SNYCQ_post-ses-02-task-CPTS_images = thoughts in the form of images

SNYCQ_post-ses-02-task-CPTS_words = thoughts in the form of words

SNYCQ_post-ses-02-task-CPTS_vague = thoughts rather specific or vague

SNYCQ_post-ses-02-task-CPTS_intrusive = self-assessed vigilance

**TAP_A: TAP-alertness**

TAP_A_1 = reaction time medians for 1. round (no signal)

TAP_A_2 = reaction time medians for 2. round (signal)

TAP_A_3 = reaction time medians for 3. round (signal)

TAP_A_4 = reaction time medians for 4. round (no signal)

*NO signal (aggregate scores)*

TAP_A_5 = mean reaction time (no signal)

TAP_A_6 = median reaction time (no signal)

TAP_A_7 = percent rank of the reaction time (no signal; compared to norm sample)

TAP_A_8 = standard deviations (no signal)

TAP_A_9 = percent rank of the standard deviation (no signal; compared to norm sample)

*Signal (aggregate scores)*

TAP_A_10 = mean reaction time (signal)

TAP_A_11 = median reaction time (signal)

TAP_A_12 = percent rank of the reaction time (signal; compared to norm sample)

TAP_A_13 = standard deviations (signal)

TAP_A_14 = percent rank of the standard deviation% (signal; compared to norm sample)

*Phasic alertness*

TAP_A_15 = phasic alertness

TAP_A_16 = percent rank of the phasic alertness (compared to norm sample)

**TPS: Tuckman Procrastination Scale**

TPS_D_sum = procrastination sum score

**AUT: Alternative Uses Task**

AUT_Fluency: fluency score (amount of generated ideas)

AUT_creative_quality: rated creative quality of generated ideas

AUT_Elaboration_mean: detailedness of generated ideas

AUT_Average_Uniqueness: statistical rarity (frequency) of generated idea

**BCQ: Body Cognition Questionnaire**

BCQ_private_body_mean: private body subscale mean score

BCQ_public_body_mean: public body subscale mean score

BCQ_body_competence_mean: body competence subscale mean score

**SYN: Synesthesia Consistency Score**

SYN_consistency: a score of 1 suggests presence of synesthesia, non-synesthetes usually score around 2 if asked to memorize color-letter association, otherwise around 3

**FFMQ: Five Facets of Mindfulness Questionnaire**

FFMQ_observe_sum: observation of internal and external processes: Non-judgement about mental processes

FFMQ_describe_sum: describing internal processes

FFMQ_act_awareness_sum: conscious actions

FFMQ_nonjudge_sum: non-judgement about mental processes

FFMQ_nonreact_sum: non-reaction on mental processes

**BDI: Beck Depressions Inventar**

BDI_sum_1 = sum of Item 1

BDI_sum_2 = sum of Item 2

BDI_sum_3 = sum of Item 3

BDI_sum_4 = sum of Item 4

BDI_sum_5 = sum of Item 5

BDI_sum_6 = sum of Item 6

BDI_sum_7 = sum of Item 7

BDI_sum_8 = sum of Item 8

BDI_sum_9 = sum of Item 9

BDI_sum_10 = sum of Item 10

BDI_sum_11 = sum of Item 11

BDI_sum_12 = sum of Item 12

BDI_sum_13 = sum of Item 13

BDI_sum_14 = sum of Item 14

BDI_sum_15 = sum of Item 15

BDI_sum_16 = sum of Item 16

BDI_sum_17 = sum of Item 17

BDI_sum_18 = sum of Item 18

BDI_sum_19 = sum of Item 19

BDI_sum_20 = sum of Item 20

BDI_sum_21 = sum of Item 21

BDI_summary_sum = sum of all items

**MCQ: Metacognition Questionnaire**

MCQ_lack_of_cogn_conf_mean: cognitive confidence (assessing confidence in attention and memory)

MCQ_pos_bel_about_worry_mean: positive beliefs about worry

MCQ_cogn_self-consc_mean: cognitive self-consciousness (the tendency to focus attention on thought processes)

MCQ_neg_bel_about_uncontr_danger_mean: negative beliefs concerning the consequences of not controlling thoughts

MCQ_need_contr_thoughts_mean: cognitive self-consciousness (the tendency to focus attention on thought processes)

**STAXI: State-Trait Anger Expression Inventor**

STAXI_anger_trait =trait anger sum score

STAXI_anger_inward = trait anger inward sum score (holding anger in)

STAXI_anger_outward = trait anger outward sum score (expressing anger)

STAXI_anger_control = trait anger control sum score (ability to control anger)

**SDS: Social Desirability Scale**

SDS_sum = social desirability sum score

**TMT: Trail Making Test**

TMT_1 = time it took to connect numbers (seconds. milliseconds)

TMT_2 = brain functions based on performance for Trail A (Reitan & Wolfson, 1988)

TMT_3 = how many errors did the pp. make

TMT_5 = time it took to connect numbers and letters (seconds. milliseconds)

TMT_6 = brain functions based on performance for Trail B (Reitan & Wolfson, 1988)

TMT_7 = how many errors did the pp. make

**S-D-MW : Spontaneous and Deliberate Mind Wandering**

S-D-MW_delib_mean = deliberate mind wandering (mean score)

S-D-MW_spont_mean = spontaneous mind wandering (mean score)

**AMAS: Abbreviated Math Anxiety Scale**

AMAS_sum = math anxiety sum scale

**IAT: Internet Addiction Test**

IAT_sum = internet addiction sum score

**PSSI: Personality Style and Disorder Inventory**

PSSI_PN = self-willed/paranoid sum scores

PSSI_SZ = reserved/schizoid sum scores

PSSI_ST = suspecting/schizotypic sum scores

PSSI_BL = spontaneous/borderline sum scores

PSSI_HI = endearing/histrionic sum scores

PSSI_NA = ambitious/narcissistic sum scores

PSSI_SU = self-critical/insecure sum scores

PSSI_AB = loyal/dependent sum scores

PSSI_ZW = careful/compulsive sum scores

PSSI_NT = critical/negativistic sum scores

PSSI_DP = quiet/depressive sum scores

PSSI_SL = helpful/selfless sum scores

PSSI_RH = optimistic/rhapsodic sum scores

PSSI_AS = autonomous/antisocial sum scores

**RAT: Remote Associates Task**

RAT_CORRECT_NR = amount of correct items

RAT_PERCENT = % correct items

RAT_Rtmeanforcorrectanswers = mean reaction time for correct answers

**TAP_I: TAP-incompatibility test**

*Compatible stimuli*

TAP_I_1 = mean time for compatible stimuli

TAP_I_2 = median time for compatible stimuli

TAP_I_3 = percent rank of the reaction time (compared to norm sample)

TAP_I_4 = standard deviations

TAP_I_5 = percent rank of the standard deviations (compared to norm sample)

TAP_I_6 = how many errors did participant make during compatible stimuli presentation

TAP_I_7 = percent rank of the errors (compared to norm sample)

*Incompatible stimuli*

TAP_I_8 = mean time for incompatible stimuli

TAP_I_9 = median time for incompatible stimuli

TAP_I_10 = percent rank of the reaction time (compared to norm sample)

TAP_I_11 = standard deviations

TAP_I_12 = = percent rank of the standard deviations (compared to norm sample)

TAP_I_13 = how many errors did participant make during incompatible stimuli presentation

TAP_I_14 = = percent rank of the error count (compared to norm sample)

*Whole stimuli*

TAP_I_15 = mean time for whole stimuli presentation

TAP_I_16 = median time for whole stimuli presentation

TAP_I_17 = percent rank of the reaction time (compared to norm sample)

TAP_I_18 = standard deviations

TAP_I_19 = percent rank of the standard deviations (compared to norm sample)

TAP_I_20 = how many errors did participant make during whole stimuli presentation

TAP_I_21 = percent rank of the error count (compared to norm sample)

*F-values*

TAP_I_22 = F-value_visual_field

TAP_I_23 = percent rank of the F-value_visual_field (compared to norm sample)

TAP_I_24 =F-value_hand (indicanting effect of handedness)

TAP_I_25 = percent rank of the F-value_hand (low rank indicates higher reaction time difference between hands; compared to norm sample)

TAP_I_26 = F-value_visual_field_x_hand

TAP_I_27 = percent rank of the F-value_visual_field_x_hand (compared to norm sample)

**UPSS: Impulsive Behavior Scale**

UPPS_Mean_NegUrg = negative urgency (mean score)

UPPS_Mean_Premed = (lack of) premeditation (mean score)

UPPS_Mean_Persev = (lack of) perseverance (mean score)

UPPS_Mean_SS = sensation seeking (mean score)

UPPS_Mean_PosUrg = positive urgency (mean score)

**SE: Self-Esteem Scale**

SE_Mean_SelfEst = self-esteem mean score

**GoldMSI: Goldsmiths Musical Sophistication Index**

GoldMSI_Active_sum = active engagement with music sum score

GoldMSI_Training_sum = musical training sum score
