# Supplementary material for: A functional connectome phenotyping dataset including cognitive state and personality measures
Source: Sci Data. 2019 Feb 12;6:180307. doi: 10.1038/sdata.2018.307 (PMC6371896; doi:10.1038/sdata.2018.307)
Supplement: Supplementary Table 2 [file sdata2018307-s4.docx]

**Supplementary Table 2. Magnetic resonance imaging exclusion criteria.**

**Max Planck Institute’s magnetic resonance imaging safety questionnaire**

| **Please tick the following as appropriate:** | **Answers** |
| --- | --- |
| Do you have a pacemaker? | O Yes O No |
| Do you have a portable insulin pump or drug pump? | O Yes O No |
| Do you have a shunt (cerebral water drainage)? | O Yes O No |
| Do you have metal clips as a result of an operation? (e.g., clips after intra-abdominal surgery, stents, coils…)? | O Yes O No |
| Do you have artificial heart valves? | O Yes O No |
| Do you have metal splints or metal objects in the body or eye? | O Yes O No |
| Do you work in the metal-processing industry? | O Yes O No |
| Do you have metal joint prostheses? | O Yes O No |
| Do you have metal plates, screws, or nails as a result of bone injuries? | O Yes O No |
| Do you have inner ear implants? | O Yes O No |
| Do you have any other metal objects (including piercings, dental braces, retainer, dental implants) on or in the body? | O Yes O No |
| Do you have implanted electrodes for deep brain stimulation or are you wearing other electronic devices on or in your body? | O Yes O No |
| Are you currently wearing hot patches or nicotine patches? | O Yes O No |
| Do you have tattoos or permanent makeup? | O Yes O No |
| Have you had an operation within the last two months?  If yes, please describe | O Yes O No |
| Do you tend to claustrophobia? | O Yes O No |
| Are you able to lie completely still on your back during the entire examination? | O Yes O No |
| Are you particularly sensitive to noise or do you suffer from tinnitus? | O Yes O No |
| Are you known to have epileptic seizures? | O Yes O No |
| Do you suffer from disturbances of the cardiac rhythm? | O Yes O No |
| Do you suffer from other heart diseases (e.g., cardiac valvular disease, coronary disease)? | O Yes O No |
| Do you suffer from vascular disorders or circulatory disturbances of the arteries? | O Yes O No |
| Do you suffer from respiratory disorders (e.g.,, acute respiratory infections, bronchial asthma, chronic bronchitis, tuberculosis)? | O Yes O No |
| Is it possible that you are pregnant? | O Yes O No |
| Do you suffer from allergic reactions? | O Yes O No |
| Do you suffer from disturbances of the kidney functions? | O Yes O No |
| Have you ever suffered from or are you currently suffering from a neurological disease (e.g., meningitis, cerebral haemorrhage, cerebral infarct)? | O Yes O No |
| Have you had injuries in the head region (e.g., concussion)? | O Yes O No |
| Do you take any kind of medication? If yes, which medication? | O Yes O No |
| Are you currently wearing an intrauterine device (“IUD, contraceptive coil”)?  If so, please confirm the following comment/explanation:  “I am aware of a possible dislocation of the IUD and consequent loss of its contraceptive effect as a result of the MRI examination and, despite that, I am willing to take part in the examination. I agree to have the position of my IUD checked by a gynecologist after the MRI examination.”  ____________________________________________  Participant’s signature – Forename, Surname | O Yes O No |
